# Supplementary material for: Basophils activate splenic B cells and dendritic cells via IL-13 signaling in acute traumatic brain injury
Source: J Neuroinflammation. 2025 Dec 17;22:290. doi: 10.1186/s12974-025-03621-1 (PMC12709802; doi:10.1186/s12974-025-03621-1)
Supplement: Supplementary file 1 — Supplementary Material 1: Supplementary Table 1: List of antibodies and lncRNA sequences. [file 12974_2025_3621_MOESM1_ESM.docx]

**Supplementary Table 1: List of antibodies and lncRNA sequences**

| **Antigen (primary antibody)** | **Catalogue number** | **Company** | **Dilution** |
| --- | --- | --- | --- |
| Guinea pig anti CD11c | HS-375 004 | Synaptic Systems | 1:500 (IF); 1:250 (ISH) |
| Rabbit anti CD19 | ab245235 | Abcam | 1:200 (IF); 1:100 (ISH) |
| Mouse anti CD19 | 14-0199-82 | Invitrogen | 1:100 |
| Rat anti CD4 | 14-9766-82 | E-bioscience | 1:100 (IF); 1:50 (ISH) |
| Rat anti MCPT8 | 647402 | Biolegend | 1:200 (IF); 1:100 (ISH) |
| Rabbit anti S6-RP (phosphor) | 2211S | CST | 1:200 (IF); 1:1000 (WB) |
| Mouse anti IL-13 | SC-393365 | Santa Cruz | 1:50 |
| Rabbit anti IL-13Ra1 (phosphor) | PA5-38607 | Invitrogen | 1:50 |
| Rabbit anti 4E-BP1 (phosphor) | 9455P | CST | 1:500 |
| Rabbit anti ERK1/2 (phosphor) | 9101S | CST | 1:1000 (WB) |
| Mouse anti beta-actin | 69009-I-Ig | Proteintech | 1:500 |
| Mouse anti SSEA-1 (CD15) | NB100-1831SS | Novus | 1:500 |
| Rabbit anti CD16 | bs-6028r | Bioss | 1:100 |
| Goat anti SiglecF | AF1706-SP | R6D systems | 1:200 |
| Mouse anti ENPP-3/CD203c | NBP1-44643 | Novus | 1:300 |
| Rabbit anti FceR1a | 10980-1-AP | Proteintech | 1:100 |
| Chicken anti MAP2 | CPCA-MAP2 | Encor | 1:500 |
|  |  |  |  |
| **Antigen (secondary antibody)** | **Catalogue number** | **Company** | **Dilution** |
| Donkey anti guinea pig 405 | ab175678 | Abcam | 1:500 |
| Goat anti guinea pig 488 | A11073 | Invitrogen | 1:500 |
| Donkey anti mouse 405 | ab175658 | Abcam | 1:500 |
| Donkey anti mouse 568 | A10037 | Invitrogen | 1:500 |
| Donkey anti mouse 647 | A31571 | Invitrogen | 1:500 |
| Donkey anti rabbit 647 | A21207 | Invitrogen | 1:500 |
| Donkey anti rabbit 568 | A10042 | Invitrogen | 1:500 |
| Donkey anti rat 488 | A21208 | Invitrogen | 1:500 |
| FluoTag-X2 anti mouse AbberriorStar 580 | N1202-Ab580-S | Nanotag | 1:500 |
| FluoTag-X4 anti Rabbit AbberiorStar 635p | N2404-Ab635P-S | Nanotag | 1:500 |

| **Gene** | **Sequence** |
| --- | --- |
| MALAT1 | forward: 5’ -atagcccaggaaagagtgcg- 3’  reverse: 5’ - gcttcaccaccacatccgta- 3’ |
| HOTAIR | forward: 5’ -gcgccaacgtagaccaaaag- 3’  reverse: 5’ -taccgatgttggggacctct- 3’ |
| CYRANO | forward: 5‘ -aggttctgtggcgtgagttg- 3‘  reverse: 5‘ -actgcggtcaactgtgctta- 3‘ |
| NORAD | forward: 5’ -tcctgagttgaccgcattgt- 3’  reverse: 5’ -ctttccactcacggaccaca- 3’ |
