# Supplementary figures and images for: Basophils activate splenic B cells and dendritic cells via IL-13 signaling in acute traumatic brain injury
Source: J Neuroinflammation. 2025 Dec 17;22:290. doi: 10.1186/s12974-025-03621-1 (PMC12709802; doi:10.1186/s12974-025-03621-1)

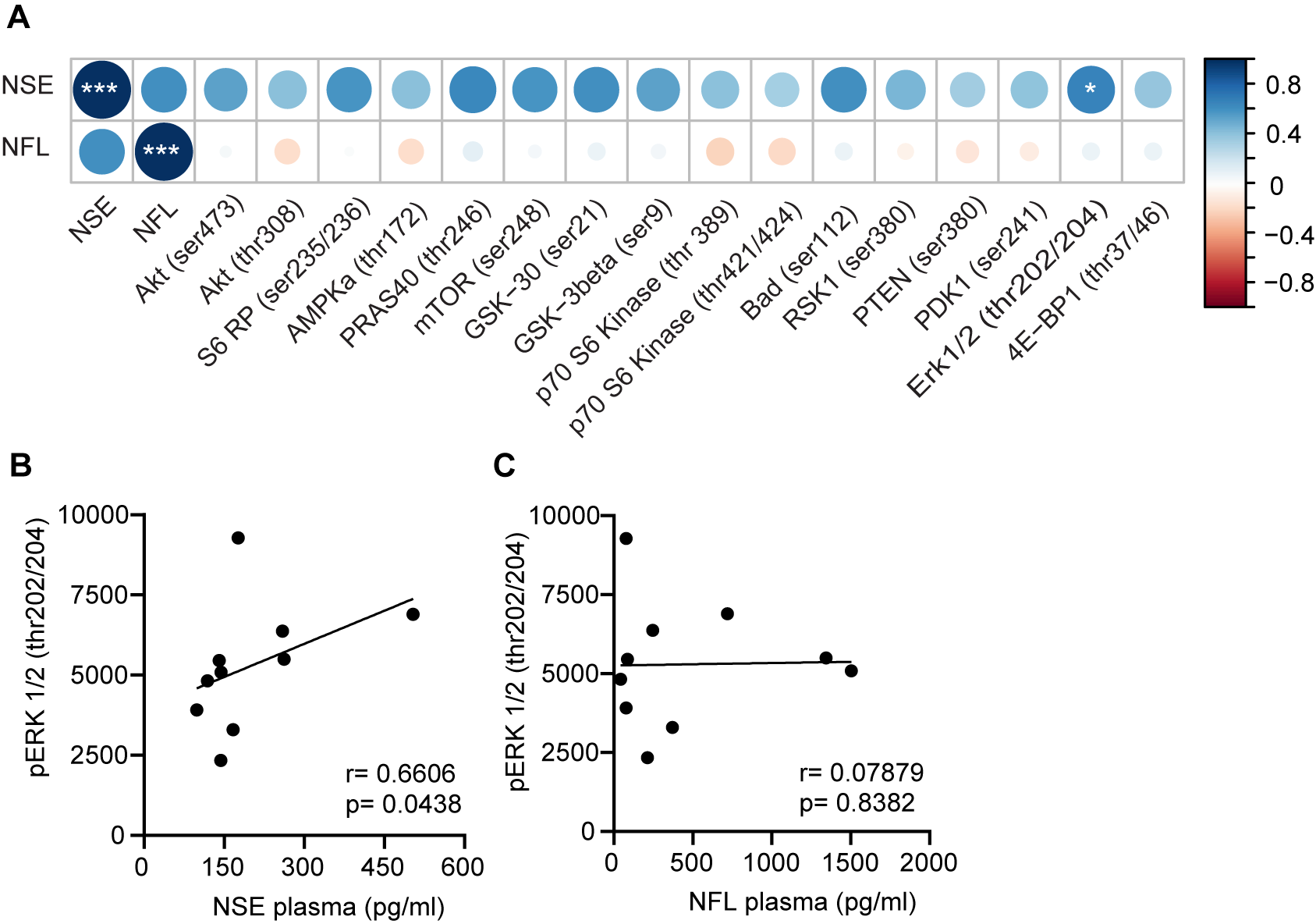

Supplement: Supplementary file 2 — Supplementary Material 2: Supplementary Figure 1: Splenic signaling correlates with neuronal damage, but not axonal damage. A. The correlation matrix reveals high R values between the signaling targets and NSE plasma values, with a significance in pERK. R values close to 0 were observed between signaling targets and NFL plasma values. B-C. Single target correlation shows the significant correlation between NSE and pERK, and poor correlation between NFL and pERK. N = 9. Data is shown as Spearman R correlation matrix, R value as color coded and p value depicted in the graph. *: p<0.05. [file 12974_2025_3621_MOESM2_ESM.tif]

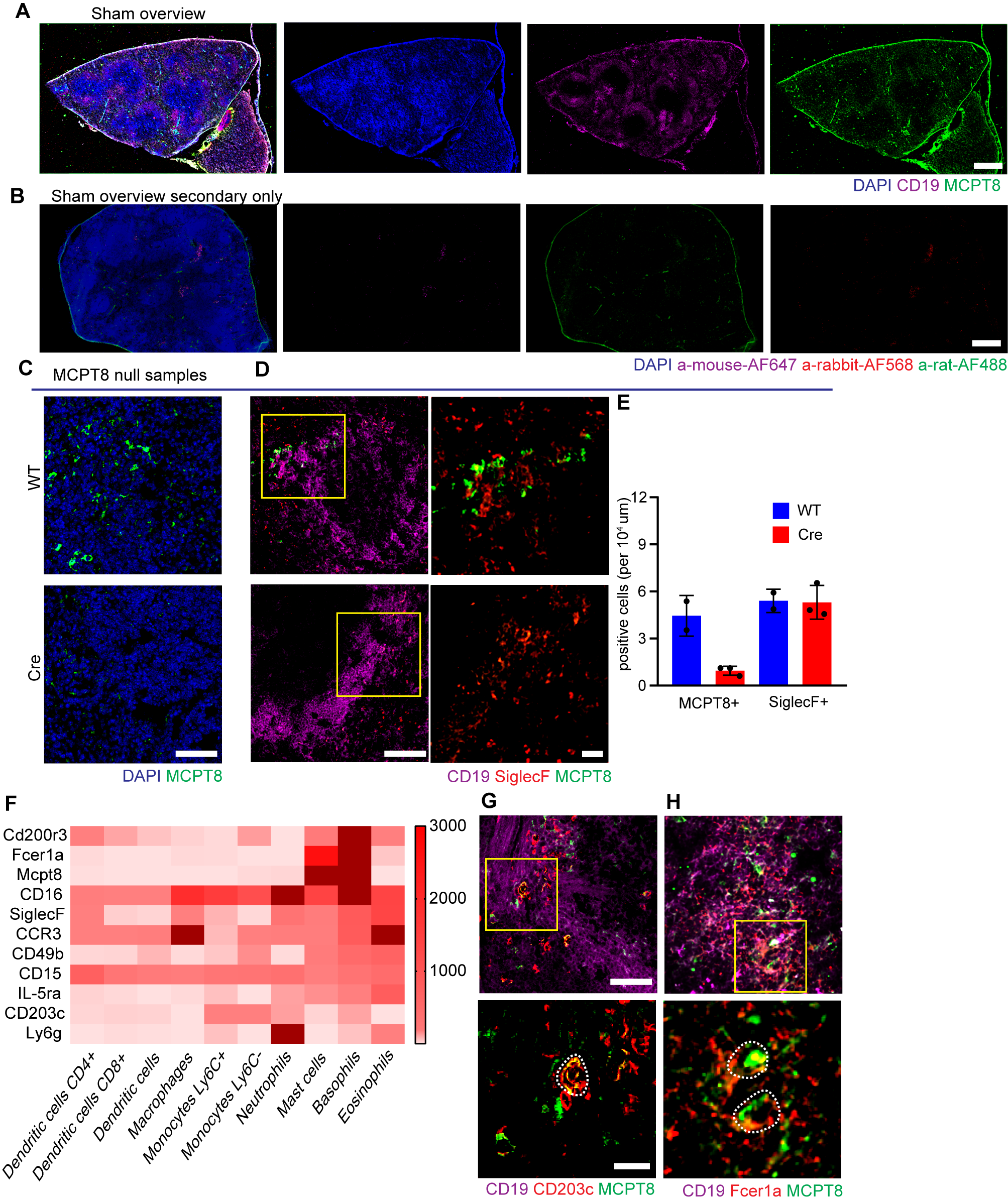

Supplement: Supplementary file 3 — Supplementary Material 3: Supplementary Figure 2: Overview images and validation of antibodies. A. Immunofluorescence staining and imaging of CD19, MCPT8 and DAPI in spleen sections showed the dishomogeneous distribution of CD19 and MCPT8. With a main localization in the white pulp, marginal zone and follicles. Scale bar 500 µm. B. Immunofluorescence staining and imaging of DAPI, a-rat AF-488, a-mouse AF-568 and a-rabbit AF 647 in spleen sections showed little to no unspecific binding of secondary antibodies to spleen tissue sections. Scale bar 500 µm. C-E. Immunofluorescence staining and imaging of in one set DAPI and MCPT8 and another set CD19, SiglecF and MCPT8 in WT and MCPT8-cresamples show a strong decrease of MCPT8+ cells in the cre samples with no differences in eosinophil marker SiglecF compared to WT control samples. Scale bar overview: 50 µm; scale bar insert: 20 µm. WT N = 2; Cre N = 3. Data is shown as mean±SD F. Heatmap with data extracted from the Immgen database, showing a selection of immune cells and expression of several granulocyte, eosinophil and basophil markers with levels of expression depicted in red. H, I. Immunostaining of spleen sections with a subset of markers with high and intermediate expression on basophils reveal a colocalization of known highly expressed basophil markers CD203c and Fcer1a with MCPT8. Scale bar overview: 50 µm; scale bar insert: 20 µm. [file 12974_2025_3621_MOESM3_ESM.tif]

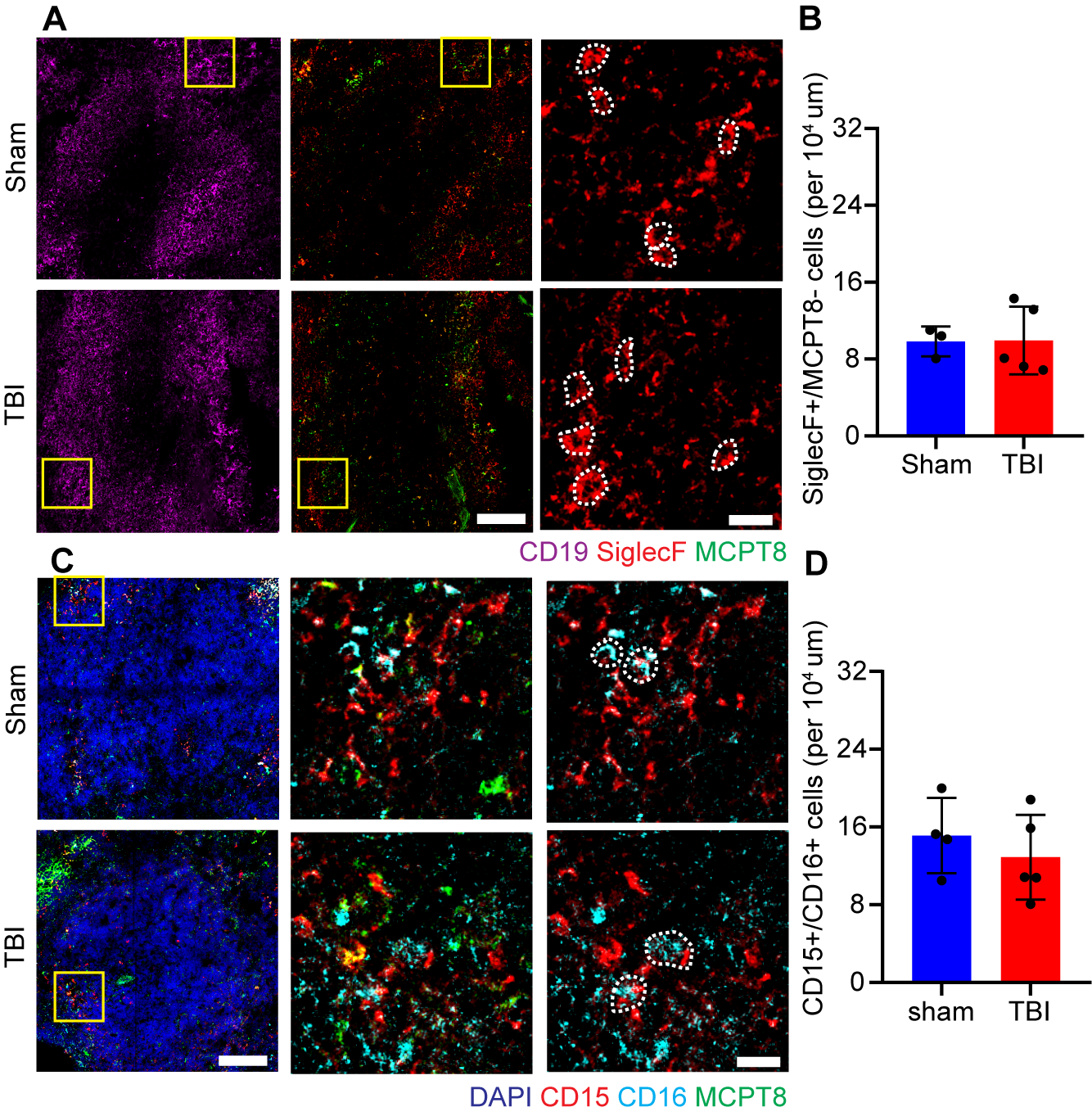

Supplement: Supplementary file 4 — Supplementary Material 4: Supplementary Figure 3: Eosinophils and Neutrophils show no difference post TBI.A-B. Immunofluorescence staining of spleen sections with CD19, SiglecF and MCPT8 show no differences in SiglecF+/MCPT8- cells3 h post TBI. Sham N = 3; TBI N = 5, scale bar overview: 100 µm; scale bar insert: 20 µm. C-D. Immunofluorescence staining of spleen sections with CD19, CD15 and CD16 show no differences in CD15+/CD16+ cells3 h post TBI. Sham N = 4; TBI N = 5, scale bar overview: 100 µm; scale bar insert: 20 µm. [file 12974_2025_3621_MOESM4_ESM.tif]

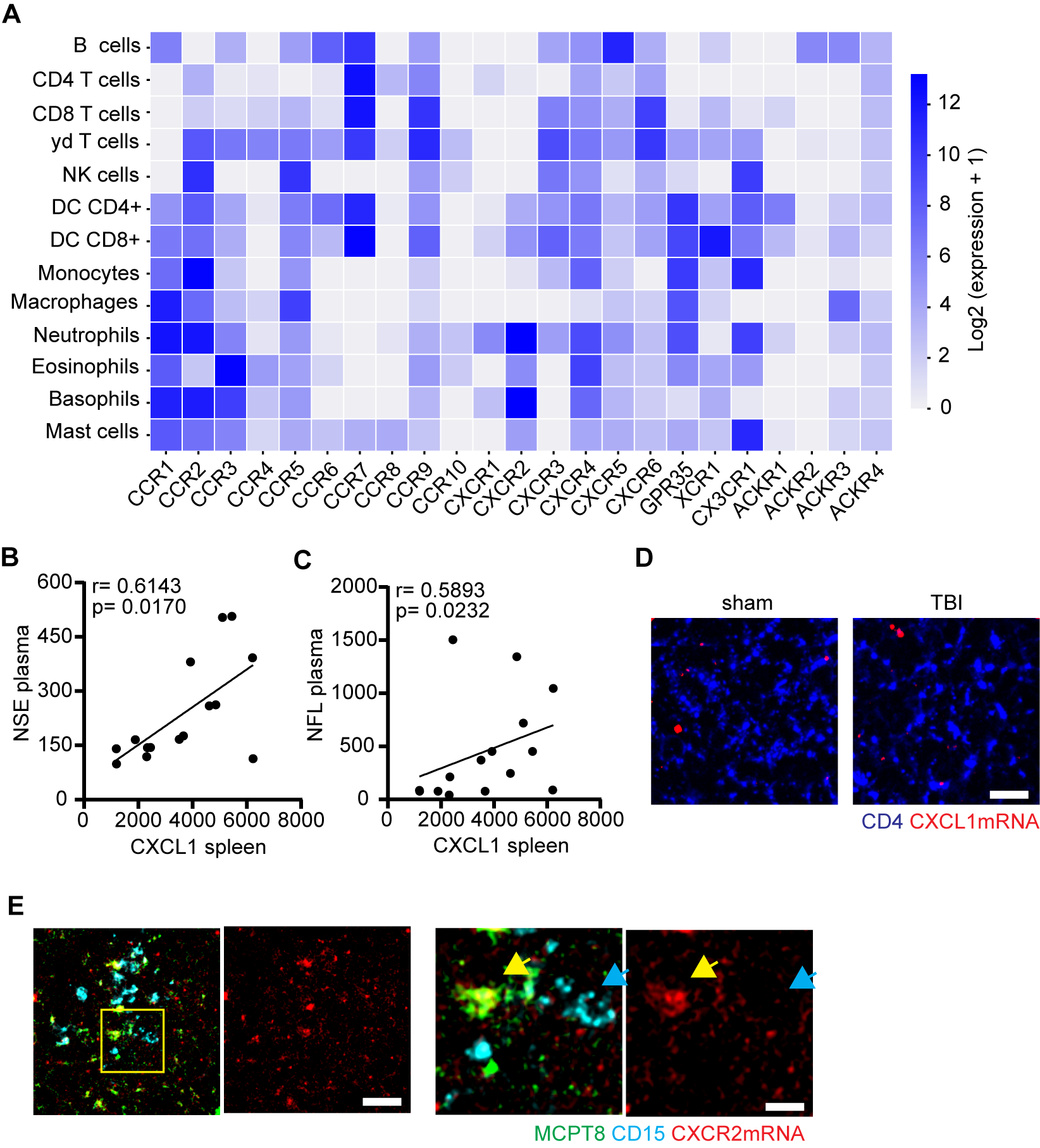

Supplement: Supplementary file 5 — Supplementary Material 5: Supplementary Figure 4: CXCL1 chemoattracts basophils via its cognate receptor CXCR2.A. Heatmap with data extracted from the Immgen database, showing a selection of immune cells and expression of most common chemokine receptors reveal a high expression of CXCR2on both basophils and neutrophils. B-C. Pearson R correlation analysis reveals a significant positive correlation between splenic CXCL1 protein levels with both NSEand NFLplasma levels. D. Single mRNA in situ hybridization of CXCL1 co-stained with CD4 showed no expression of CXCL1 in CD4+ cells in either sham nor TBI. N = 5. scale bar: 10 µm. E. Single mRNA in situ hybridization of CXCR2 co-stained with MCPT8 and CD15 showed high expression of CXCR2 in MCPT8+ cellsand lower expression in CD15+ cellsN = 3. Scale bar overview: 50 µm; scale bar insert: 10 µm. [file 12974_2025_3621_MOESM5_ESM.tif]

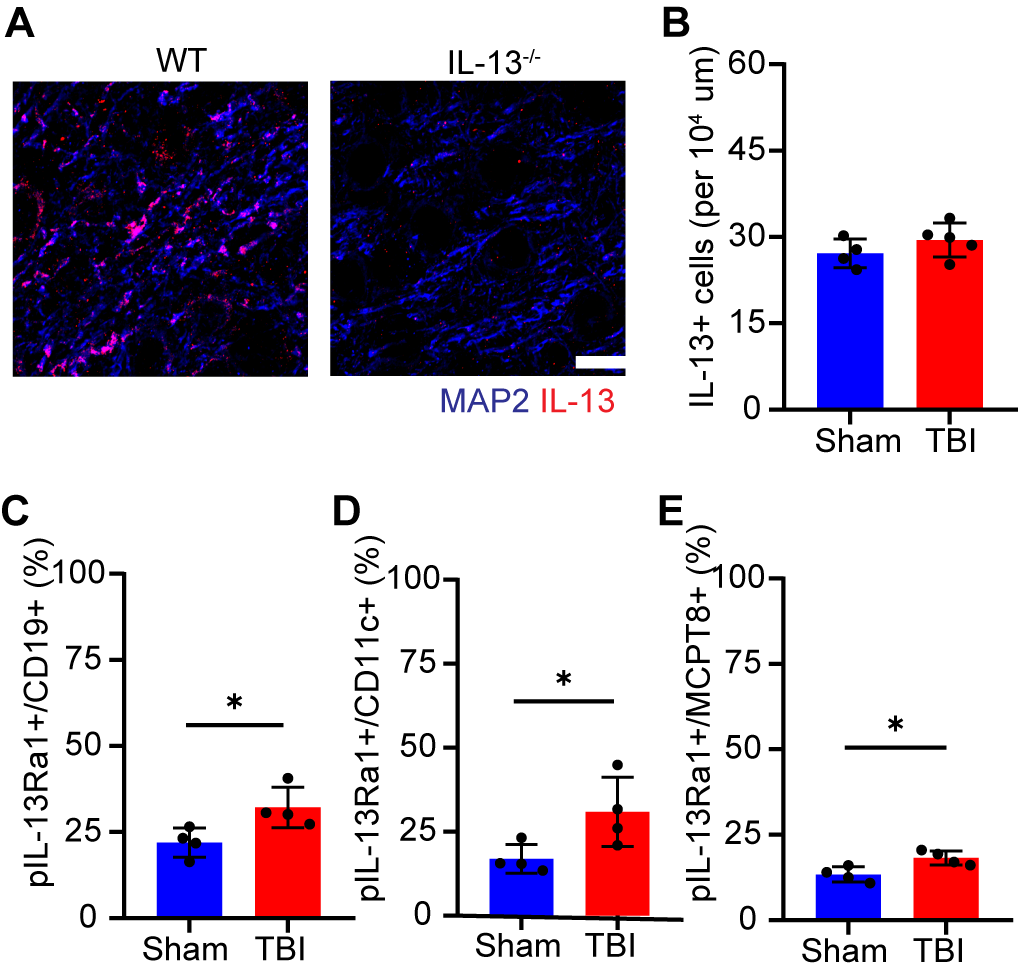

Supplement: Supplementary file 6 — Supplementary Material 6: Supplementary Figure 5: Basophils activate B-cells and DCs via IL-13Ra1 phosphorylation.A. Immunofluorescence staining of brain tissue with MAP2 and IL-13 in WT and IL-13-/- samples reveal IL-13 antibody specificity. N = 3. Scale bar: 10 µm. B. Analysis of immunofluorescence staining of spleen sections with IL-13 shows no difference in the number of IL-13+ cells post TBI. Sham N = 4; TBI N = 5. C-E. Analysis of the immunofluorescence staining of spleen sections with CD19, CD11c, MCPT8 and pIL-13Ra1, depicted in barplots. Corresponding to Figure 4D. N = 4. Data is shown as mean±SD. *: p < 0.05. [file 12974_2025_3621_MOESM6_ESM.tif]

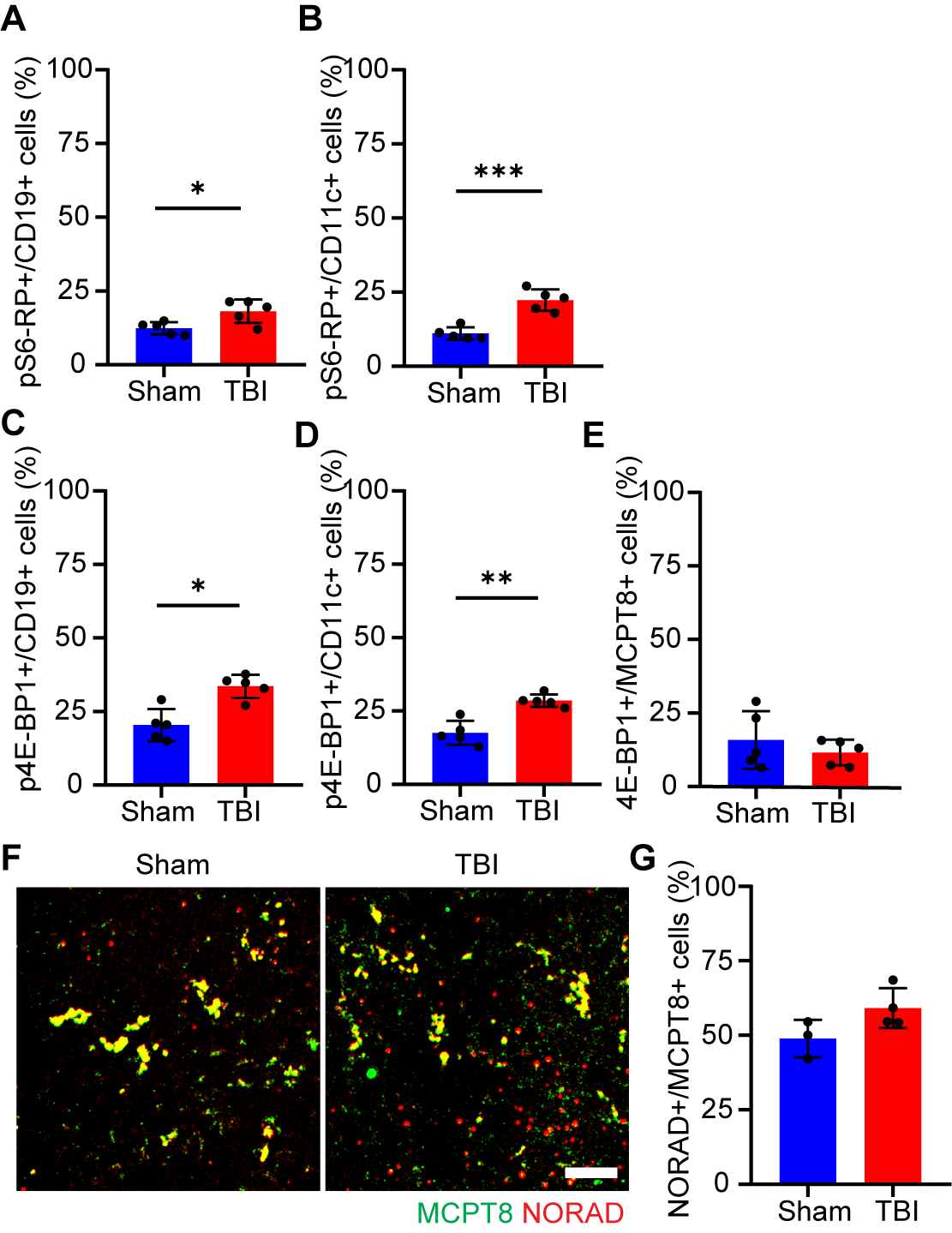

Supplement: Supplementary file 7 — Supplementary Material 7: Supplementary Figure 6: Basophils induce fast translational response in B-cells and DCs post TBI.A, B. Analysis of the immunofluorescence staining of spleen sections with CD19, CD11c and pS6-RP, depicted in barplots. Corresponding to the representative images in Figure 6A. N = 5. C-E. Analysis of the immunofluorescence staining of spleen sections with CD19, CD11c, MCPT8 and p4E-BP1, depicted in barplots. Corresponding to the representative images in Figure 6C. N = 5. F, G. Single lncRNA RNAscope in spleen sections with the probe NORAD and a co-staining with MCPT8 revealed a high amount of NORAD in MCPT8+ cells, with no significant differences between sham and TBI. Sham N = 3; TBI N = 4; scale bar insert: 20 µm. Data is shown as mean±SD. *: p < 0.05, **: p<0.01. [file 12974_2025_3621_MOESM7_ESM.tif]

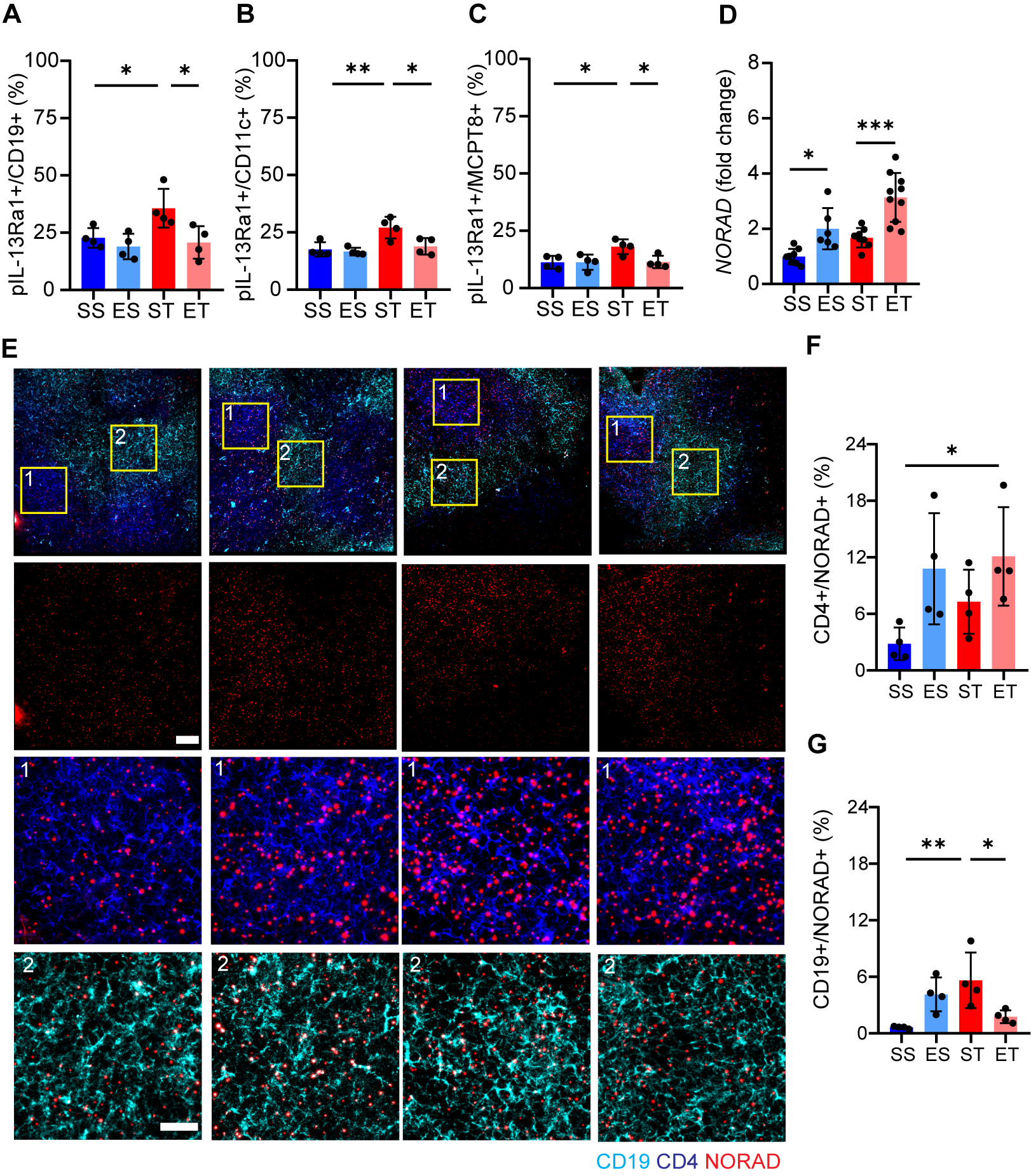

Supplement: Supplementary file 8 — Supplementary Material 8: Supplementary Figure 7: Ethanol pretreatment prevents basophil induced IL-13Ra1 phosphorylation and NORAD expression, B-cells and DCs.A-C. Analysis of the immunofluorescence staining of spleen sections with CD19, CD11c, MCPT8 and pIL-13Ra1, depicted in barplots. Corresponding to the representative images in Figure 8D. N = 4. D. Qualitative RT-PCR in spleen samples revealed a significant increase in NORAD upon ethanol pretreatmentpost TBI. SS N = 8; ES N = 6; ST N = 8; ET N = 10. E-G. Single lncRNA RNAscope in spleen sections with the probe NORAD and a co-staining with CD19 and CD4 revealed a significant increase in CD19+/NORAD+ upon TBI, with a decrease upon ethanol pretreatment. CD4+ cells show a steady increase in NORAD+ with a significant increase between sham and ethanol pretreated TBI. SS N = 4; ES N = 4; ST N = 4; ET N = 4, scale bar overview: 50 µm; scale bar insert: 20 µm. Data is shown as mean±SD. *: p < 0.05, **: p<0.01, ***: p<0.001. [file 12974_2025_3621_MOESM8_ESM.tif]

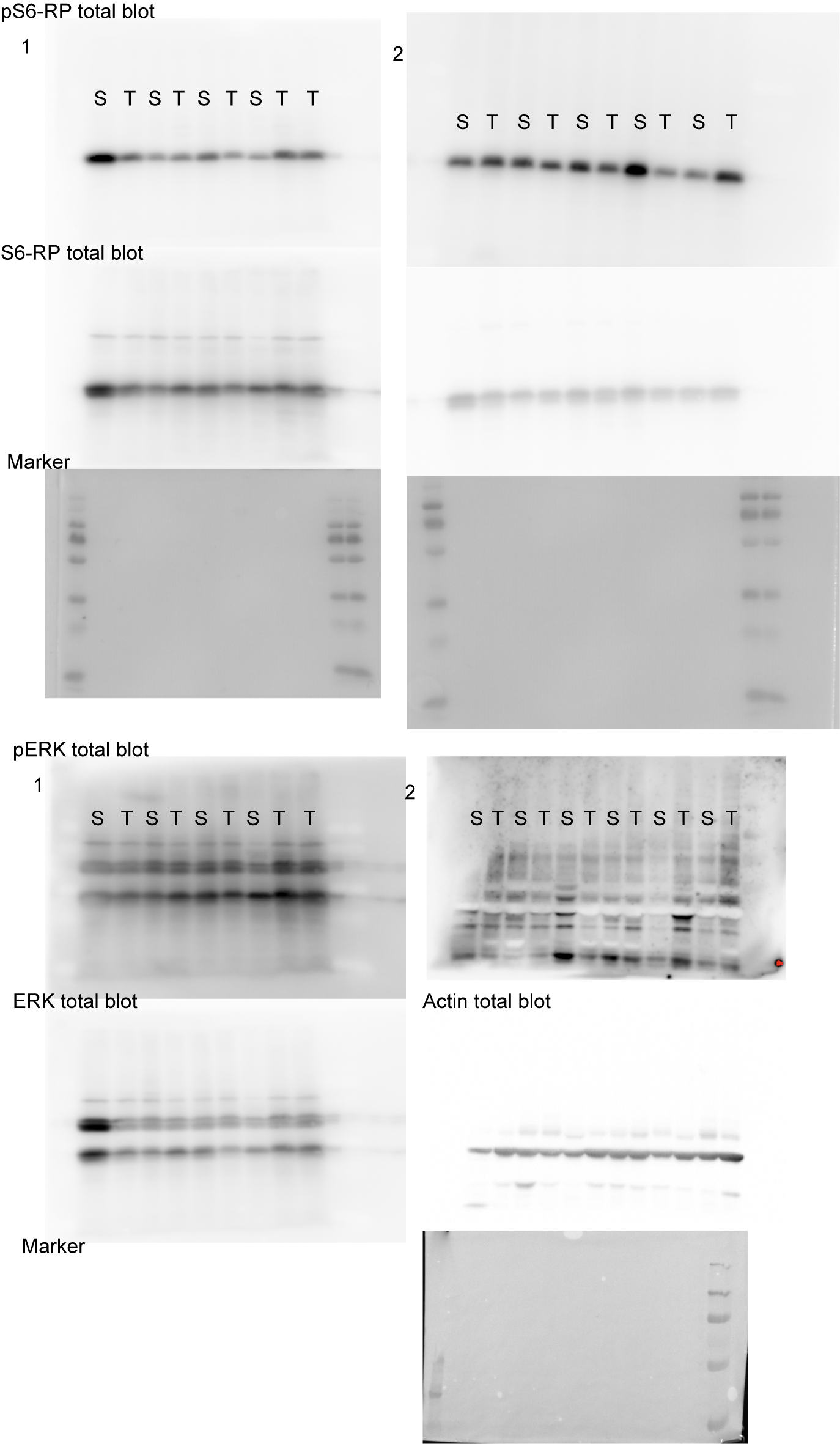

Supplement: Supplementary file 9 — Supplementary Material 9 [file 12974_2025_3621_MOESM9_ESM.tif]
